# Serum metabolomics profiling by proton nuclear magnetic resonance spectroscopy reveals sexual dimorphism and masculinization of intermediate metabolism in women with polycystic ovary syndrome (PCOS)

**DOI:** 10.1186/s13293-023-00507-w

**Published:** 2023-04-19

**Authors:** Héctor F. Escobar-Morreale, M. Ángeles Martínez-García, María Insenser, Nicolau Cañellas, Xavier Correig, Manuel Luque-Ramírez

**Affiliations:** 1 Diabetes Obesity and Human Reproduction Research Group, Hospital Universitario Ramón y Cajal, Universidad de Alcalá, Centro de Investigación Biomédica en Red Diabetes y Enfermedades Metabólicas Asociadas (CIBERDEM), Instituto Ramón y Cajal de Investigación Sanitaria (IRYCIS), Carretera de Colmenar km 9.1, 28034 Madrid, Spain; 2 Department of Electronic Engineering, Centro de Investigación Biomédica en Red Diabetes y Enfermedades Metabólicas Asociadas (CIBERDEM), Institut d’Investigació Sanitària Pere Virgili, University Rovira i Virgili, Tarragona, Spain

**Keywords:** Androgens, Estrogens, Metabolism, Obesity, Polycystic ovary syndrome, Sex

## Abstract

**Background:**

The polycystic ovary syndrome (PCOS) is associated with insulin resistance, obesity and cardiometabolic comorbidities. We here challenged the hypothesis, using state-of-the art proton nuclear magnetic resonance spectroscopy metabolomics profiling, that androgen excess in women induces also a certain masculinization of intermediate metabolism that is modulated by obesity.

**Methods:**

Participants were 53 Caucasian young adults, including 17 women with classic PCOS consisting of hyperandrogenism and ovulatory dysfunction, 17 non-hyperandrogenic women presenting with regular menses, and 19 healthy men, selected in order to be similar in terms of age and body mass index (BMI). Half of the subjects had obesity defined by a body mass index ≥ 30 kg/m^2^. Subjects maintained the same diet unrestricted in carbohydrates for 3 days before sampling and maintained their lifestyle and exercise patterns prior and during the study. Plasma samples were submitted to proton nuclear magnetic resonance spectroscopy metabolomics profiling.

**Results:**

Obesity associated a metabolomics profile mainly characterized by increased branched chain and aromatic aminoacids. Regardless of obesity, this unfavorable profile also characterized men as compared with control women, and was shared by women with PCOS. Notably, the negative impact of obesity on metabolomics profile was restricted to women, with obese men showing no further deterioration when compared with their non-obese counterparts.

**Conclusions:**

Serum metabolomics profiling by proton nuclear magnetic resonance spectroscopy reveals sexual dimorphism, and masculinization of intermediate metabolism in women with PCOS, further suggesting a role for sex and sex hormones in the regulation of intermediate metabolism.

## Background

The polycystic ovary syndrome (PCOS) is characterized by a combination of symptoms and signs that includes androgen excess and/or ovarian dysfunction in the form of oligo-anovulation and/or polycystic ovaries [1]. PCOS is frequently associated with disorders of intermediate metabolism such as visceral adiposity, obesity and type 2 diabetes [2–4].

Fifteen years ago we proposed that women with PCOS suffer from a vicious circle from early ages whereby androgen excess favoring the abdominal deposition of fat further facilitates androgen secretion by the ovaries and adrenals [5]. Insulin resistance and compensatory hyperinsulinism facilitates ovarian androgen excess because insulin acts as a co-gonadotropin at the ovary [6]. But also, hyperandrogenism facilitates a predominantly visceral deposition of body fat [7] and adipose tissue dysfunction [8–11], further contributing to insulin resistance and hyperinsulinism [5]. The serum gas chromatography–mass spectrometry metabolomics phenotype of people with PCOS indicates a major role of obesity in the metabolic associations of this syndrome: while non-obese women with PCOS show evidence of central (hepatic) insulin resistance, peripheral insulin sensitivity is conserved in this subset of patients, whereas adipose and muscle insulin resistance only develops in obese patients [12].

Aside from obesity, androgen excess may play a role in the metabolic derangements of PCOS. Such a role is supported by the existence of sexual dimorphism in fasting and postprandial metabolism [13], with men usually showing worse metabolic profiles than women that are exaggerated by obesity [14–16]. Accordingly, our earlier studies showed evidence for masculinization of both adipose tissue distribution [2] and function [8–11] in women with PCOS. Hence, sexual dimorphism might be also present in fasting metabolomics profiles and, if sex hormones play a role in such sex differences, the profiles of women with PCOS should resemble those of men to some extent.

## Materials and methods

### Aim of the study

We here challenged the hypothesis, using state-of-the art proton nuclear magnetic resonance (^1^H-NMR) spectroscopy metabolomics profiling, of the existence of sexual dimorphism in the fasting metabolomic profiles of young adults, and that androgen excess in women may induce a certain masculinization of intermediate metabolism, with obesity exerting a modifying role.

### Subjects

This report is part of a broader study addressing postprandial changes in hormonal profiles, metabolic mediators and markers of oxidative stress and inflammation in young adults (PI11/00357). Thus, a detailed description of phenotyping, protocol and methodology has been described elsewhere [16–23].

The study included 53 Caucasian young adults: 17 women with PCOS, 17 non-hyperandrogenic women presenting with regular menses, and 19 healthy men, selected in order to be similar in terms of age and body mass index (BMI). We classified individuals into non-obese (BMI < 30 kg/m^2^, n = 28) and obese (BMI ≥ 30 kg/m^2^, n = 25) subgroups. Total body fat mass was estimated using a body fat monitor (Omron BF 300, Omron Corp., Kyoto, Japan) and was expressed as kg and percentage of total body mass. All patients met the National Institutes of Health 1990 criteria [24] for the diagnosis of PCOS, requiring the presence of the classic phenotype consisting of clinical and/or biochemical hyperandrogenism, oligo/anovulation, and exclusion of secondary etiologies such as hyperprolactinemia, nonclassic congenital adrenal hyperplasia and hypothyroidism. We did not include non-hyperandrogenic phenotypes of PCOS because the study aimed to address the metabolic effects of androgen excess in women. Control women and men had no history of hypogonadism (including male obesity-associated secondary hypogonadism), infertility or menstrual dysfunction, and none subject presented smoking habits or had received treatment with oral contraceptives, antiandrogens, sex steroids, insulin sensitizers or drugs that might interfere with clinical or biochemical variables for at least 6 months before sampling. Three men, two women with PCOS and one control woman reported having used antibiotics within 3 months prior to recruitment. In women, hirsutism was quantified using the modified Ferriman–Gallwey score [25]. PCOS was ruled out in the control women because all of them presented without menstrual and ovulatory dysfunction and had no evidence of clinical and biochemical androgen excess. The female and male control groups were composed of healthy volunteers recruited from the hospital’s staff and of overweight or obese people seeking medical advice at our Department.

### Assays

All women were evaluated during the follicular phase of the menstrual cycle or in amenorrhea, after excluding pregnancy. Patients were instructed to follow the same diet—containing at least 300 g of carbohydrates per day—for 3 days before sampling in order to avoid false positive results in the 75 g oral glucose tolerance test (OGTT), which was used not only for research purposes but also to check the patients for disorders of glucose tolerance. Plasma samples were obtained after a 12 h overnight fast, processed and frozen at − 20 °C or − 80 °C. Samples were assayed for total testosterone (T), sex hormone binding globulin (SHBG), total estradiol (E_2_), androstenedione (A4), dehydroepiandrosterone-sulfate (DHEAS), 17-hydroxyprogesterone, follicle-stimulating hormone, luteinizing hormone, prolactin, thyrotropin, prolactin, insulin, glucose, and high-sensitivity C-reactive protein (hsCRP).

Serum glucose was measured by using the glucose oxidase method (Beckman Instruments, Indianapolis IN), and insulin was measured by an automated immunochemiluminescence method (Immulite 2000, Siemens Healthcare Sector, Erlangen, Germany). Total T was measured by direct radioimmunoassay (Spectria Testosterone RIA, Orion Diagnostica Oy, Espoo, Finland) and E_2_, SHBG, A4, DHEAS and hsCRP were measured using an automated immunochemiluminescence method (Immulite 2000, Siemens Healthcare Sector, Erlangen, Germany). The mean intraassay and interassay coefficients of variation were less than 10% for all these assays [26, 27]. Insulin and glucose levels at fasting and during the OGTT were used to calculate homeostasis model assessment of insulin resistance (HOMA-IR) [28] and the composite insulin sensitivity index (ISI) [29], respectively. Free T and E_2 _levels were calculated from their total concentrations and sex hormone-binding globulin (SHBG) levels, and the free T to free E_2_ molar ratio was calculated [30].

### Proton nuclear magnetic resonance spectroscopy metabolomics profiling

Serum samples were subjected to a deproteinization process to remove high-molecular-weight species. Briefly, after thawing serum samples on ice, 300 µl of each serum sample were mixed with 1400 µl of MeOH:H_2_O (8:1) and 150 µl of IS solution in 2 ml microcentrifuge tubes, incubated at − 20 °C for 20 min and centrifuged at 15,000 rpm, at 4 °C for 10 min. After centrifugation, 1300 µl of supernatant were transferred into a 2 ml microcentrifuge tube, dried under vacuum and lyophilized overnight.

Lyophilized samples were reconstituted with 600 µl of 50 mM phosphate buffer solution (PBS) with 0.05 M TSP as internal standard, and then transferred to nuclear magnetic resonance (NMR) tube for NMR analysis. A Bruker 600 MHz Spectrometer (Bruker Biospin, Rheinstetten, Germany), was used to acquire high-resolution ^1^H-NMR spectroscopy data of low molecular weight metabolites (LMWM), as sugars and amino acids, using 1D Carr-Purcell-Meiboom-Gill sequence (CPMG), with pre-saturation to suppress the residual water peak. The acquired CPMG data was phased, baseline-corrected, and referenced to the chemical shift of the α-glucose anomeric proton doublet taken at 5.233 ppm, as proposed by Pearce et al. [31]. CPMG data was used for the profiling of 36 metabolites, based on a new, fully automated version of the software package Dolphin [32]. Each metabolite was identified by checking for all its resonances along the spectra, and then quantified using line-shape fitting methods on one of its signals. Signal annotation was based on templates prepared in previous studies with the help of available databases [33] and bibliography [34–36]. Validation of metabolite identification was assisted by statistical total correlation spectroscopy [37]. Results are expressed as arbitrary units.

### Statistical analysis

Being this study part of a broader project addressing postprandial metabolism changes as a whole, sample size calculation was based on previous data of Gonzalez et al. [38] reporting differences between patients with PCOS and control women in the percentage change of nuclear factor kappaB expression in mononuclear cells after a standard oral glucose tolerance test. We used the online sample size and power calculator from the Institut Municipal d'Investigació Mèdica (Barcelona, Spain, version 7.12; https://www.imim.cat/ofertadeserveis/software-public/granmo/). Setting alpha at 0.05 and beta at 0.2 for a two-sided test, the inclusion of 8 individuals per group would allow detecting a mean difference in percentage change of 50.35%, assuming a standard deviation of 34.1%.

Data are expressed as mean ± SD (tables) or mean ± SEM (figures). Normality of continuous variables was evaluated by the Kolmogorov–Smirnov test and logarithmic transformations were applied as needed. Two-way univariate General Linear Models (GLM) were used to analyze differences in fasting variables considering group of subjects (control women, women with PCOS and men), obesity, and their interaction within a single analysis. The least significant difference post hoc test was used for multiple comparisons. Correlations between continuous variables were analyzed by Pearson’s, Spearman’s, and partial correlation analysis, as appropriate. We used SPSS Statistics 15.0 (SPSS Inc., Chicago, IL, USA) and considered as statistically significant two-tailed p-values < 0.05.

## Results

### Baseline characteristics of study subjects

The clinical, hormonal, and metabolic characteristics of participants at fasting are shown in the Table 1. As expected from design, age and BMI were not different among groups. According to sex, men showed higher total and free T levels, waist circumference (WC), waist to hip ratio (WHR) and fat mass than both groups of women, but lower levels of E_2_ and SHBG. Women with PCOS had higher hirsutism scores and circulating androgens than non-hyperandrogenic control women, but showed no statistically significant differences in terms of WC or WHR. In addition, we did not observe significant differences in metabolic parameters between men and women, or control women and participants with PCOS, with the exception of lower mean HDL-cholesterol values in men. Obese individuals, regardless of sex and PCOS status, showed increased total and free E_2_ values, free T, fasting glucose, insulin, and HOMA-IR, and decreased ISI and SHBG concentrations.

**Table 1:** Clinical, metabolic and hormonal variables in control women, patients with PCOS and men

	Control women		Women with PCOS		Control men		Group	Obesity	Interaction
	Non-obese (n = 9)	Obese (n = 8)	Non-obese (n = 9)	Obese (n = 8)	Non-obese (n = 10)	Obese (n = 9)	*P*	*P*	*P*
Age (years)	26 ± 5	27 ± 6	24 ± 8	30 ± 5	24 ± 4	25 ± 4	0.342	0.101	0.319
Body mass index (kg/m^2^)	23 ± 2	36 ± 4	24 ± 3	37 ± 5	23 ± 2	34 ± 3	0.226	**< 0.001**	0.782
Waist circumference (cm)^a,b^	76 ± 9	100 ± 17	72 ± 7	105 ± 11	81 ± 5	110 ± 13	0.050	**< 0.001**	0.377
Waist-to-hip ratio^a,b^	0.75 ± 0.08	0.83 ± 0.12	0.73 ± 0.05	0.85 ± 0.06	0.83 ± 0.04	0.90 ± 0.05	**0.002**	**< 0.001**	0.436
Fat mass (kg)^a,b^	22.5 ± 4.8	42.5 ± 10.3	20.2 ± 5.5	43.7 ± 8.6	12.9 ± 4.5	36.7 ± 12.6	**0.011**	**< 0.001**	0.767
Fat mass (%)^a,b^	35.0 ± 5.3	43.7 ± 5.7	31.6 ± 6.3	42.9 ± 3.9	16.6 ± 5.1	32.6 ± 7.0	**< 0.001**	**< 0.001**	0.165
Hirsutism score	1.4 ± 1.3	1.8 ± 1.2	9.7 ± 4.5	9.3 ± 4.5	–	–	**< 0.001**	0.738	0.876
Total T (nmol/l)^a,b,c^	1.6 ± 0.3	2.0 ± 0.5	2.5 ± 0.7	2.4 ± 1.0	18.5 ± 3.3	17.3 ± 3.6	**< 0.001**	0.776	0.196
Total E_2_ (pmol/l)^a,b^	149 ± 63	276 ± 200	182 ± 201	149 ± 49	68 ± 16	94 ± 26	**< 0.001**	**0.024**	0.422
Free T (pmol/l)^a,b,c^	21 ± 7	31 ± 8	36 ± 12	45 ± 24	450 ± 104	464 ± 94	**< 0.001**	**0.024**	0.265
Free E_2_ (pmol/l)^a^	2.7 ± 1.1	5.3 ± 3	3.6 ± 4.3	3.4 ± 1.4	1.8 ± 0.5	2.6 ± 0.7	**0.010**	**0.003**	0.520
Free T/free E_2_^a,b^^,c^	8.6 ± 0.9	7.6 ± 1.6	17.4 ± 4.0	14.0 ± 1.9	263.4 ± 22.6	186.4 ± 12.7	**< 0.001**	0.119	0.750
SHBG (nmol/l)^a,b^	56 ± 25	43 ± 14	50 ± 21	32 ± 13	27 ± 10	20 ± 6	**< 0.001**	**0.008**	0.568
Androstenedione (nmol/l)^b,c^	9.1 ± 2.9	9.5 ± 2.9	14.7 ± 4.2	13.4 ± 6.1	7.1 ± 1.7	9.3 ± 3.8	**< 0.001**	0.712	0.371
hsCRP (nmol/l)	27 ± 22	38 ± 28	20 ± 21	65 ± 74	31 ± 25	31 ± 12	0.998	**0.009**	0.207
Triglycerides (mmol/l)	0.84 ± 0.36	0.92 ± 0.34	0.90 ± 0.25	1.14 ± 0.47	0.89 ± 0.29	1.15 ± 0.40	0.399	0.054	0.711
Total cholesterol (mmol/l)	4.4 ± 0.9	4.7 ± 0.9	4.3 ± 1.0	4.4 ± 0.9	4.1 ± 0.6	4.8 ± 0.9	0.844	0.133	0.485
HDL-cholesterol (mmol/l)^a,b^	1.4 ± 0.3	1.3 ± 0.2	1.4 ± 0.2	1.2 ± 0.2	1.2 ± 0.2	1.0 ± 0.1	**0.001**	**0.002**	0.512
LDL-cholesterol (mmol/l)	2.7 ± 0.8	2.9 ± 0.6	2.5 ± 0.8	2.7 ± 0.8	2.4 ± 0.4	3.3 ± 0.8	0.507	**0.034**	0.237
Fasting insulin (pmol/l)	55 ± 20	77 ± 21	52 ± 29	94 ± 25	40 ± 11	75 ± 27	0.123	**< 0.001**	0.440
Fasting glucose (mmol/l)^b,c^	4.7 ± 0.4	5.3 ± 0.4	4.5 ± 0.5	4.8 ± 0.5	4.9 ± 0.5	5.2 ± 0.4	**0.011**	**0.001**	0.408
Insulin sensitivity index	6.6 ± 2.8	3.3 ± 1.2	8.1 ± 4.7	3.6 ± 1.4	7.3 ± 2.8	3.8 ± 1.6	0.640	**< 0.001**	0.909
HOMA-IR	1.6 ± 0.6	2.6 ± 0.7	1.5 ± 0.9	2.9 ± 0.7	1.3 ± 0.4	2.5 ± 1.0	0.422	**< 0.001**	0.744

Data are means ± SD. The effects of group and obesity were analyzed by a two-way GLM followed by the least significant difference *post-hoc* test

*E*_*2*_ estradiol, *HDL* high-density lipoprotein, *LDL* low-density lipoprotein, *HOMA-IR* homeostasis model assessment of insulin resistance, *hsCRP* high-sensitivity C-reactive protein, *PCOS* polycystic ovary syndrome, *SHBG* sex hormone-binding globulin, *T* testosterone

^a^*p* < 0.05 for the difference between men and control women regardless of obesity

^b^*p* < 0.05 for the difference between men and PCOS women regardless of obesity

^c^*p* < 0.05 for the difference between PCOS and control women regardless of obesity. *P*-values < 0.05 are indicated in bold

### Differences between groups of subjects

**Fig. 1:**
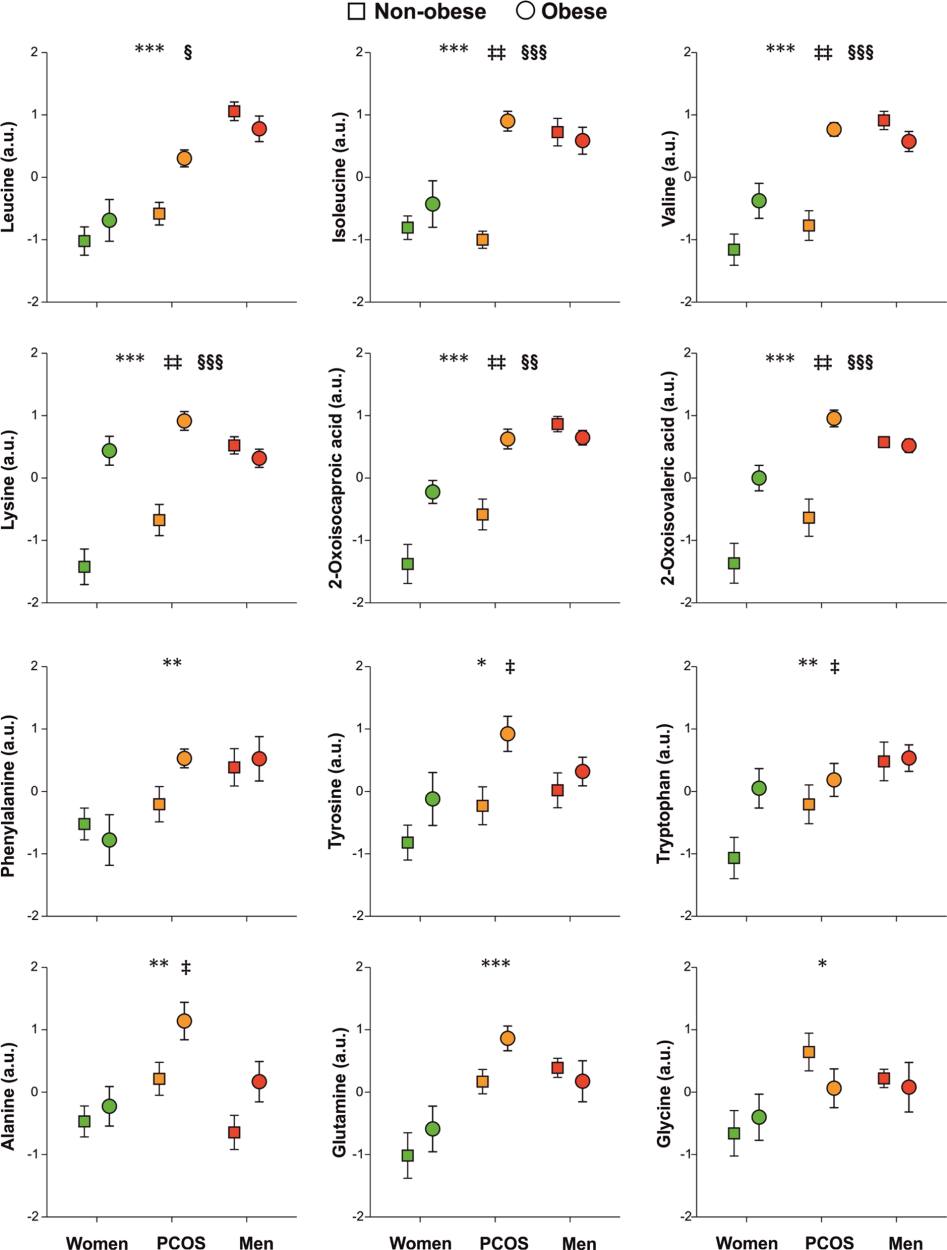
Proton nuclear magnetic resonance spectroscopy metabolomics profiling of branched-chain and aromatic amino acids, and other amino acids and by-products as a function of group of subjects, obesity and, their interaction. Data are arbitrary units and are expressed as means ± SEM. Green symbols are control women; orange symbols are people with PCOS; and red symbols are control men. Squares are non-obese subjects and circles are obese people. *p < 0.05, **p < 0.01 and ***p < 0.001 for the differences among the groups of subjects regardless of obesity; ^‡^*p* < 0.05, ^‡‡^*p* < 0.01 and ^‡‡‡^*p* < 0.001 for the differences among non-obese and obese people, regardless of the group of subjects; ^§^*p* < 0.05, ^§§^*p* < 0.01 and ^§§§^*p* < 0.001 for the interaction between obesity and group of subjects

**Fig. 2:**
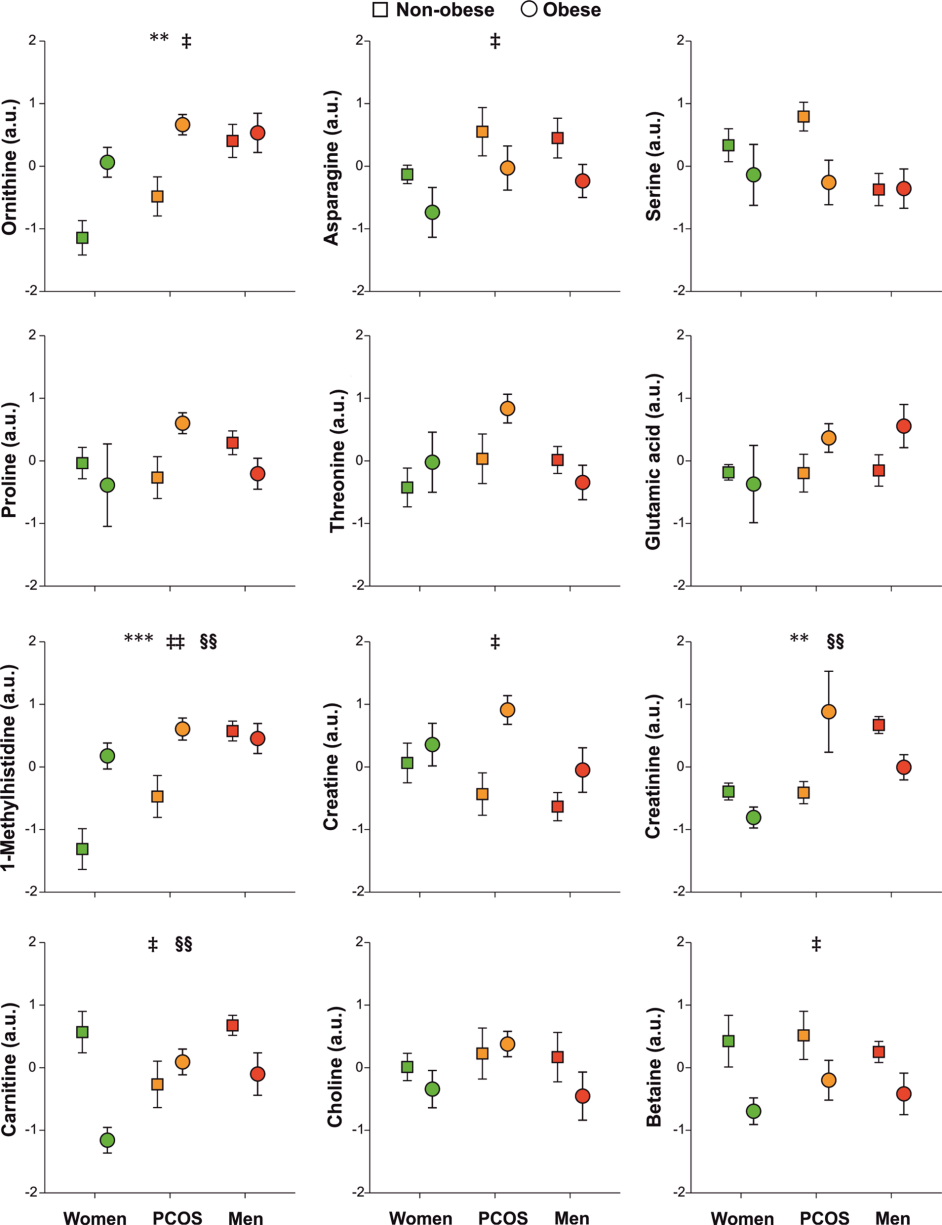
Proton nuclear magnetic resonance spectroscopy metabolomics profiling of other amino acids and derived metabolites as a function of group of subjects, obesity, and their interaction. Data are arbitrary units and are expressed as means ± SEM. Green symbols are control women; orange symbols are people with PCOS and red symbols are control men. Squares are non-obese subjects and circles are obese people. *p < 0.05, **p < 0.01 and ***p < 0.001 for the differences among groups of subjects regardless of obesity; ^‡^*p* < 0.05, ^‡‡^*p* < 0.01 and ^‡‡‡^*p* < 0.001 for the differences among non-obese and obese people, regardless of the group of subjects; ^§^*p* < 0.05, ^§§^*p* < 0.01 and ^§§§^*p* < 0.001 for the interaction between obesity and group of subjects

**Fig. 3:**
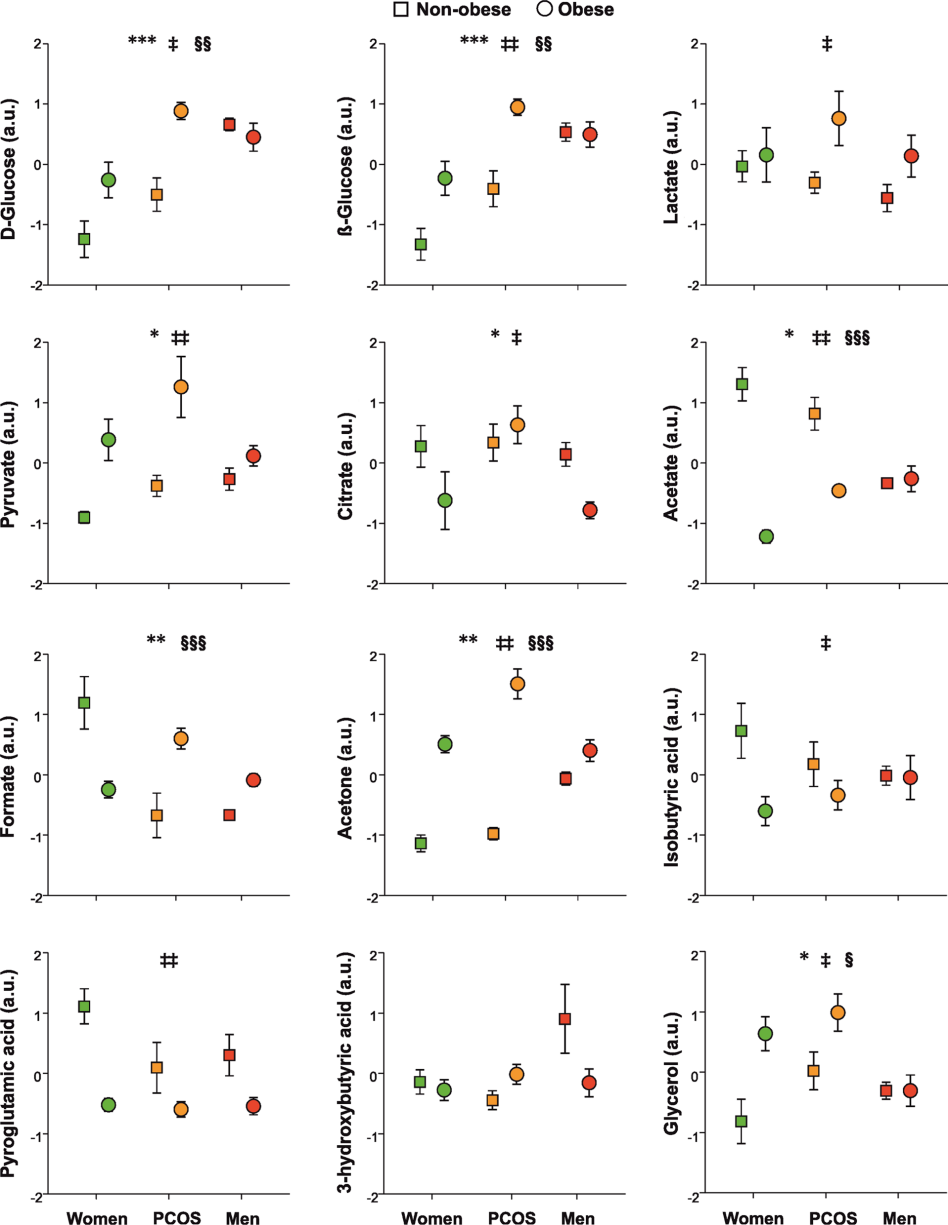
Proton nuclear magnetic resonance spectroscopy metabolomics profiling of carbohydrates, short-chain fatty acids, ketone bodies, and glycerol, as a function of group of subjects, obesity, and their interaction. Data are arbitrary units and are expressed as means ± SEM. Green symbols are control women; orange symbols are people with PCOS; and red symbols are control men. Squares are non-obese subjects and circles are obese people. *p < 0.05, **p < 0.01 and ***p < 0.001 for the differences among groups of subjects regardless of obesity; ^‡^*p* < 0.05, ^‡‡^*p* < 0.01 and ^‡‡‡^*p* < 0.001 for the differences among non-obese and obese people, regardless of the group of subjects; ^§^*p* < 0.05, ^§§^*p* < 0.01 and ^§§§^*p* < 0.001 for the interaction between obesity and group of subjects

Our current metabolomics data supports sexual dimorphism in intermediate metabolism. The metabolomics profiles of young men resembled those previously described in association with metabolic disorders such as obesity and type 2 diabetes [39, 40]. Compared with control women, men presented with increased levels of numerous amino acids including branched-chain amino acids (BCAA: leucine, isoleucine, valine), aromatic amino acids (AAA; phenylalanine, tyrosine, tryptophan), lysine, glutamine, ornithine, and amino acid-derived metabolites such as 1-methylhistidine, creatinine, and the ketoacids 2-oxoisocaproic and 2-oxoisovaleric that derive from BCAA catabolism. Carbohydrates such as d- and β-glucose, and acetone were also augmented in men compared with control women (Figs. 1, 2, 3). Deviation from this proposedly unfavorable metabolically profiles included glycine, which is usually decreased in metabolic disorders [39, 40] but was slightly increased in men compared with women (Fig. 1) and, conversely, acetate and formate, which are usually increased in type 2 diabetes [40] but were reduced in men compared with control women in our series (Fig. 3).

Furthermore, our data suggests that androgen excess may result into masculinization of metabolomics profiles in women: people with PCOS shared with men the increase, respect to control women, in leucine, isoleucine, valine, lysine, phenylalanine, tyrosine, tryptophan, glutamine, ornithine, methylhistidine, 2-oxoisocaproic and 2-oxoisovaleric acids, d- and β-glucose, acetate and pyruvate (Figs. 1, 2, 3). In fact, levels of phenylalanine, tyrosine, glutamine, ornithine, glycine, acetone and pyruvate were actually indistinguishable from those of men (Figs. 1, 2, 3). Furthermore, women with PCOS presented levels of alanine, glycerol, citrate and creatinine that were increased compared both with control men and control women (Figs. 1 and 3).

### Impact of obesity on metabolomics profiles

#### Differences between non-obese and obese people regardless of sex and PCOS

Our series of young obese people presented, when compared with young non-obese subjects, a metabolomics profile consisting of increased of branched and aromatic aminoacids such as isoleucine, valine, alanine, lysine, tyrosine, tryptophan, ornithine, methylhistidine, creatine, glycerol, 2-oxoisocaproic and 2-oxoisovaleric acids, d- and β-glucose, lactate, acetone and pyruvate and decreased betaine, carnitine, citrate, acetate, asparagine and isobutyric and pyroglutamic acids (Figs. 1, 2, 3). We also observed near-significant trends towards increased leucine (*P* = 0.076, Fig. 1) and decreased serine (*P* = 0.059, Fig. 2) in obese persons compared with non-obese counterparts.

#### Interaction of obesity with groups of subjects

Our GLM analysis also permitted to find if the effects of obesity on metabolomics profiles were specific of control women, people with PCOS and/or men, and viceversa, as indicated by a statistically significant interaction between obesity and groups of subjects. Such an analysis revealed a common pattern of changes consisting of obesity increasing or decreasing the levels of several metabolites—leucine, isoleucine, valine, lysine, methylhistidine, serine, 2-oxoisocaproic and 2-oxoisovaleric acids, glycerol, acetate, formate, d- and β-glucose, acetone—described above in both control women and patients with PCOS, but such an unfavorable effect of obesity was not found in obese men (Figs. 1, 2). Considering this interaction, and also the independent effects of PCOS described above, many of these metabolites reached levels in obese women with PCOS that were similar, or even surpassed, those of lean and obese men (Figs. 1, 2, 3).

Finally, Table 2 provides a summary of the effects of obesity, sex, PCOS and their interactions on the levels of the metabolites studied here.

**Table 2:** Summary of the effects of obesity, sex, PCOS, and their interaction on serum metabolomics profiles as assessed by ^1^H-NMR spectroscopy

Metabolite	Obesity	Sex (M vs. W)	PCOS (PCOS vs. W)	Interaction
Branched-chain amino acids and degradation products, aromatic amino acids				
Leucine	ns	↑	↑	↑ with obesity only in PCOS
Isoleucine	↑	↑	↑	↑ with obesity only in PCOS
Valine	↑	↑	↑	↑ with obesity but not in men
2-Oxoisocaproic acid	↑	↑	↑	↑ with obesity but not in men
2-Oxoisovaleric acid	↑	↑	↑	↑ with obesity but not in men
Phenylalanine	ns	↑	↑	ns
Tyrosine	↑	↑	↑	ns
Tryptophan	↑	↑	ns	ns
Other amino acids				
Lysine	↑	↑	↑	↑ with obesity but not in men
Alanine	↑	ns	↑	ns
Glutamine	ns	↑	↑	ns
Glycine	ns	↑	↑	ns
Ornithine	↑	↑	↑	ns
Asparagine	↓	ns	ns	ns
Glutamic acid	ns	ns	ns	ns
Serine	ns	ns	ns	ns
Proline	ns	ns	ns	ns
Threonine	ns	ns	ns	ns
Amino acid-derived metabolites				
1-Methylhistidine	↑	↑	↑	↑ with obesity but not in men
Pyroglutamic acid	↓	ns	ns	ns
Creatine	↑	ns	ns	ns
Creatinine	ns	↑	↑	↑ with obesity only in PCOS
Carnitine	↓	ns	ns	↓ with obesity but not in PCOS
Choline	ns	ns	ns	ns
Betaine	↓	ns	ns	ns
Carbohydrates, short-chain fatty acids, ketone bodies, and glycerol				
d-Glucose	↑	↑	↑	↑ with obesity but not in men
β-Glucose	↑	↑	↑	↑ with obesity but not in men
Lactate	↑	ns	ns	ns
Pyruvate	↑	ns	↑	ns
Citrate	↓	ns	↑	ns
Acetate	↓	↓	ns	↓ with obesity but not in men
Formate	ns	↓	↓	↓ in obese women ↑ in obese PCOS
Isobutyric acid	↓	ns	ns	ns
3-Hydroxybutyric acid	ns	ns	ns	ns
Acetone	↑	↑	↑	↑ only in non-obese men ↑ only in obese PCOS
Glycerol	↑	ns	↑	↑ with obesity but not in men

Only effects reaching statistical significance are shown

↑: higher; ↓: lower; M: control men; ns: not significant; PCOS: women with polycystic ovary syndrome; W: control women

## Discussion

Our present results not only confirm the impact of obesity on the metabolome, but also reveal sexual dimorphism that, as supported by the masculinization of metabolomics profiles of women with androgen excess, possibly implicates sex steroids and, particularly, androgens.

In our series of young adults, obesity associated a pattern of metabolic dysregulation—consisting of the increase of branched and aromatic aminoacids, and several byproducts of their metabolism—that has been previously associated with obesity, insulin resistance, the metabolic syndrome and type 2 diabetes [39–43]. The underlying mechanisms might be bidirectional, with leucine-mediated activation of the mammalian target of rapamycin complex 1 resulting in uncoupling of insulin signaling at an early stage and accumulation of mitotoxic metabolites of branched aminoacids promoting β-cell mitochondrial dysfunction, stress signaling and apoptosis associated with diabetes, and insulin resistance favoring aminoacidemia by increasing the protein degradation that insulin normally suppresses, and/or impairing oxidative metabolism of branched aminoacids in some tissues [44].

Our results also indicate sexual dimorphism on the fasting plasma metabolomics profiles of these otherwise healthy young people, with men presenting with increased levels of many of the branched and aromatic aminoacids that characterize obesity-associated metabolic dysfunction in humans. Such sexual dimorphism did not appear as a consequence of any direct association with insulin resistance, obesity or carbohydrate metabolism, because the female and male controls studied here showed no differences in BMI, fasting glucose and insulin concentrations and surrogate indexes of insulin resistance. Moreover, serum lipid profiles were similar with the expected and the lower HDL-cholesterol concentrations of men [23, 45]. Therefore, other factors such as genetic differences, androgen and estrogen concentrations, body composition (such as the WHR and the amount of fat mass that, regardless of obesity, were increased in the male controls in our study), or even gut microbiota [43], might underlay these sex differences in metabolomics profiles. Of note, production of branched aminoacids by the microbiota has been proposed to contribute to the metabolomics profile of obesity [43, 46], and an earlier report of this series of young adults found sex-related differences in gut microbiota composition, consisting of reduced bacterial ɑ-diversity in men compared with women [47].

The findings from the hyperandrogenic women with PCOS studied here further support a role of androgens and their balance with estrogens on the metabolomics profiles of young adults. Aside from a larger increase in alanine, glycerol and citrate when compared with male and female controls, which may suggest increased transamination [41] and impaired suppression of lipolysis in adipose tissue [48] because of insulin resistance, patients with PCOS shared with men the metabolomics profiles typical of insulin resistance and obesity described above, suggesting to some extent the masculinization of metabolic function. To this regards, the increase in creatinine in women with PCOS, shared with control men, could derive from their larger muscle mass compared with control women [49], since serum androgens correlate with muscle mass in women with PCOS [50].

The fact that features of intermediate metabolism of women with PCOS resemble those of men even more closely than that of control women, has been also supported by other studies from our group using transcriptomics, lipidomics and proteomics of plasma, adipose tissue and skeletal muscle [8–11, 23, 51]. Whether such a masculinization derives from direct effects of androgens and their imbalance with estrogens, or simply arise from the changes in body composition that characterize PCOS [1, 2, 5], are beyond the scope of the present study. Moreover, differences in the genotype and genetic regulators such as non-coding RNAs, including the long non-coding RNA X-inactive specific transcript, might also contribute both to sexual dimorphism and sex-biased disorders [52, 53] such as PCOS [9, 54–56].

Interestingly, sex and sex hormones influenced markedly the impact of obesity on metabolomics profiles: for many of the metabolites previously associated with metabolic dysfunction, obesity worsened these profiles in women, but did not result into any worsening on top of the already unfavorable findings of non-obese men. In other words, obesity worsened metabolomics profiles in control and PCOS women—to the extent that the latter resembled men when obesity was present—but men appeared somehow to be protected from the effects of obesity on the metabolome.

To this regards, a few years back we hypothesized that the most beneficial adipose tissue distribution and function is that of normal women, who have low androgen and high estrogen concentrations [14]. Any imbalance favoring an increase in androgen levels in women, and the very high androgen levels characteristic of healthy men, influence adipose tissue distribution and function. Accordingly, sex steroids determine a favorable (female) or unfavorable (male) body fat distribution and function [14]. However, sex hormones also provide defensive mechanisms against visceral fat accumulation: estrogens determine the metabolically safer deposition of body fat into the subcutaneous gluteal–femoral depot in women, whereas androgens increase lean and muscle mass in men, decreasing the amount of visceral fat relative to total body mass and its negative consequences [14]. Women with PCOS, on the contrary, might suffer the deleterious effects of androgens without the benefit of a larger muscle mass, facilitating the development of metabolic dysfunction.

Even though similar considerations might apply to our present metabolomics findings, an alternative explanation to the lack of worsening of metabolomics profiles in the obese men in our series would derive from our present experimental design, in which hypogonadal men were excluded because we included male subjects to serve as a completely androgenized control group. Yet the gonadal dysfunction characteristic of obesity in men is male obesity-associated secondary hypogonadism [14, 57, 58], a disorder that associates exactly the same cardiometabolic comorbidities that characterize PCOS in women [14]. A posteriori, we may speculate if any worsening of metabolomics profiles would have been found had we included obese men with secondary hypogonadism among our male controls, yet the answer to this question remains open.

Our study is not free from several limitations. The sample size of our subgroups was relatively small and was calculated with another objective in mind, thus precluding detection of smaller effects of obesity, sex and PCOS on the metabolome. Moreover, unlike earlier studies, our results derive from a population of young healthy adults in whom overt metabolic dysfunction is rare, and the women with PCOS in our series showed the classic hyperandrogenic phenotype, precluding extrapolation of the results to milder non-hyperandrogenic phenotypes of the syndrome. Women with PCOS differed from controls mostly in their hyperandrogenic background but not in abnormalities of carbohydrate metabolism or lipid profiles. Also, we could not standardize diet for more than 3 days before sampling and did not use food diaries to address long-term differences in diet among the subjects; these as factors may impact gut microbiota, which is a major contributor to the metabolome. Despite these shortcomings, the study was compensated by the homogeneous population studied in terms of age, BMI and metabolic dysfunction, a recommendation to follow the same diet for 3 days before sampling, and the use of state-of-the art metabolomics techniques.

## Perspectives and significance/conclusions

In summary, serum metabolomics profiling by ^1^H-NMR spectroscopy reveals sexual dimorphism, and masculinization of intermediate metabolism in women with PCOS, further suggesting a role for sex and sex hormones in the regulation of intermediate metabolism.

## Data Availability

The datasets used and/or analysed during the current study are available from the corresponding author on reasonable request.
